# Nanoparticle Activation Methods in Cancer Treatment

**DOI:** 10.3390/biom9050202

**Published:** 2019-05-24

**Authors:** Benjamin D White, Chengchen Duan, Helen E Townley

**Affiliations:** 1 Department of Engineering Science, Oxford University, Parks Road, Oxford OX1 3PJ, UK; Benjamin.White@seh.ox.ac.uk; 2 Nuffield Department of Women’s and Reproductive Health, Oxford University John Radcliffe Hospital, Headington, Oxford OX3 9DU, UK; Chengchen.Duan@wolfson.ox.ac.uk

**Keywords:** cancer, tumour, activation, nanosystems, temporal, spatial, extrinsic, intrinsic

## Abstract

In this review, we intend to highlight the progress which has been made in recent years around different types of smart activation nanosystems for cancer treatment. Conventional treatment methods, such as chemotherapy or radiotherapy, suffer from a lack of specific targeting and consequent off-target effects. This has led to the development of smart nanosystems which can effect specific regional and temporal activation. In this review, we will discuss the different methodologies which have been designed to permit activation at the tumour site. These can be divided into mechanisms which take advantage of the differences between healthy cells and cancer cells to trigger activation, and those which activate by a mechanism extrinsic to the cell or tumour environment.

## 1. Introduction

Activation, or release of a compound, at a tumour site can mitigate the side effects often experienced during cancer treatment by localizing the treatment. In addition, localized action can also permit the use of larger effective doses at the tumour site which would not be tolerated if administered systemically. However, controlling the release of a compound, or the activity of a molecule or nanoparticle, requires the design of smart systems. Such systems can be controlled either by differences between cancerous and normal cells, or by activation from a source outside of the cell. This review first discusses the activation of nanoparticle systems due to altered cancer cell metabolism (Section 1), and discusses pH-, enzymatic-, and concentration-dependent activation. Subsequently, extrinsic activation (Section 2) is considered, which includes ultrasound, magnetic, and light and X-ray activation. 

### 1.1. Intrinsic Activation Due to Altered Cancer Cell Metabolism 

In healthy cells the intracellular pH is tightly regulated, and kept near neutral by virtue of ion transport proteins in the plasma membrane [1]. In contrast, cancer cells have an increased intracellular pH (~7.3–7.6 versus ~7.2) and a decreased extracellular pH (~6.8–7.0 versus 7.4). This acidic extracellular pH is considered a major feature of tumour tissue and is primarily due to the secretion of lactate from anaerobic glycolysis. The acidic extracellular pH activates lysosomal enzymes that have an optimal activity in this range, and also the expression of genes of pro-metastatic factors [2]. Therefore, proteins and enzymes will be differentially expressed in cancerous cells and tissues. 

#### 1.1.1. pH-Activated Nanoparticles for Cancer Treatments

Due to the differences in pH between healthy and cancerous cells, systems can be developed where changes in acidity trigger release. Release can also be triggered at a specific location in the body relating to physiological changes in pH. 

##### – Organ Specific Release

The different organs of the body naturally differ in pH, for instance the stomach (pH 1.5–3.5) [3] or intestines (pH 5.1–7.8) [4,5]. However, oral administration is particularly challenging due to the extremes in acidity, and most compounds need protection. Without protection, or controlled release, the lifetime of the drug is likely to be low due to denaturation by the highly acidic environment in the stomach [6]. This can result in concentrations at the target site being lower than needed, leading to multi-drug resistance [7,8]. However, a specifically pH-triggered release system in the intestine could be beneficial. Indeed, a solid lipid nanoparticle has been developed to only release drug at intestinal pH in the presence of specific pepsin and pancreatic enzymes. Furthermore, several different polymeric materials, such as poly(lactic-co-glycolic-acid) (PLGA) [9] and poly(acrylic acid) (PAA) [10], have been shown to result in sufficient concentrations of anti-tumour substances via oral-delivery. Tian et al. [10] reported a specific example using a PAA coated mesoporous silica nanoparticle (MSNPs) system for the oral delivery of doxorubicin (DOX). pH-responsive PAA outer-layers were used to enfold the drug-loaded silica core and to protect the cargo from the strongly acidic environment in the stomach. Only when the nanosystem reached the higher pH of the colon, would the PAA coat degrade and release the chemotherapeutic from the core. The loading capacity of PAA-coated MSNPs could reach 785.7 mg DOX per 1g of PAA/MSNPs. Incubation of human mesenchymal stem cells (hMSCs) with 300 μg mL^−1^ of the loaded PAA/MSNPs showed cytotoxicity of over 30% after 24 h [10]. 

Oral systems have also been developed for the delivery of natural products, such as curcumin which has been known to have anti-cancer effects for many years. The insolubility of curcumin (which is at ng level) greatly restricts the bioavailability by oral administration [11]. Cui et al. developed a unique self-microemulsifying drug delivery system (SMEDDS) which could significantly increase the water solubility of curcumin and enhance the adsorption rate in gastrointestinal tract by pH-responsive release. The adsorption rate increased to over 90% within 12 h compared with only 20% for free curcumin [12].

##### – Tissue Specific Release

As discussed above, it is known that tumour tissue can have a much lower microenvironmental pH than normal tissues [13,14]. This could be exploited to allow smart nano-size pH-activated systems to only release a cargo after entering the tumour tissues. This could reduce side effects and increase the accuracy of tumour targeting [13,14,15]. This system could be partly activated by the acidic tumour microenvironment (pH ~ 6.0–6.5) or cellular lysosomal pH (below 6.0). As the nanosystem enters the slightly acidic tumour microenvironment, the PEG-PY system (PEG-b-poly(carbonate) appended with pyrrolidin-1-yl-ethylamine side chains) which carries the GDC 0449 is degraded and the drug released. However, the PEG-DB system (PEG-b-poly(carbonate) appended with N,N’dibutylethylenediamine side chains) which carries the gemcitabine (GEM) collapses rapidly but only at lower cellular lysosomal pH; thereby releasing the GEM inside the cancer cells. As a result of the multi-release, the drug-loaded nanosystem could achieve much higher cytotoxicity compared with free drugs [16]. Similarly, Qian et al. reported pH-responsive nanomicelles (NM) which could both induce and monitor cellular apoptosis. With the help of two functional peptides on the surface of the NM, the system is in a stealth mode before entering the tumour and “switched on” immediately after accumulating in the acidic microenvironment of the tumour. Released TAT peptide (transactivator of transcription; GRKKRRQRRRPQ) could greatly enhance the penetration of the NM system. Furthermore, the STP peptide (pH-triggered targetting peptide; SKDEEWHKNNFPLSPG) which is connected with an aggregation-induced emission (AIE) module is activated at the initiation of apoptosis and fluoresces. In an in vivo experiment using HT-29 tumour-bearing mice, the STD-NM system (i.e., the nano-micelle with peptide functionalization and AIE module) carrying an anti-cancer drug could significantly suppress tumour growth; tumours in the control (PBS) group were approximately 800 mm^3^ compared to the treatment group which were below 200 mm^3^. Furthermore, genetic profile data revealed that the STD-NM system greatly increased the expression of apoptosis genes such as DAPK2, and suppressed anti-apoptosis genes such as IGF1 [17].

##### – Cell Specific Release

The pH of the cytoplasm in cancer cells is higher than that of healthy cells. However, within the cells the lysosomal compartments behave differently. Lysosomes in normal cells maintain an acidic environment in the range 4.5–6.5, whereas lysosomes in malignant cells can be below pH 4.5. In highly metabolic cells the lysosomes can be in the range pH 3.8–4.7 [18]. The difference between lysosomes in healthy and malignant cells makes them a good target for responsive release [19]. 

pH-sensitive polymers, metals, and lipid-based nanomaterials have become the most popular materials for the intracellular release of treatment compounds. In addition to controlling release, the coating materials sometimes also act as protection for the cargo, such as RNAi which can easily be degraded within the lysosome [20]. pH-sensitive nanovalves (NV) have been applied for sealing the cargo inside the pores of the mesoporous silica nanoparticles (MSNs) prior to delivery. The NV was composed of α-cyclodextrin (α-CD) complexed with an aniline-based stalk on the surface of MSNs. After entering cells, the lysosomal acidic pH activates the nanovalves by decreasing the bond between the stalk and α-CD and leads to the responsive release of anti-cancer drugs. Doxorubicin loaded NV-MSNs have shown good cellular toxicity by killing over 80% of MIA PaCa-2 pancreatic cancer cells compared with the same concentration of empty control NV-MSNs [21]. 

**Figure 1:**
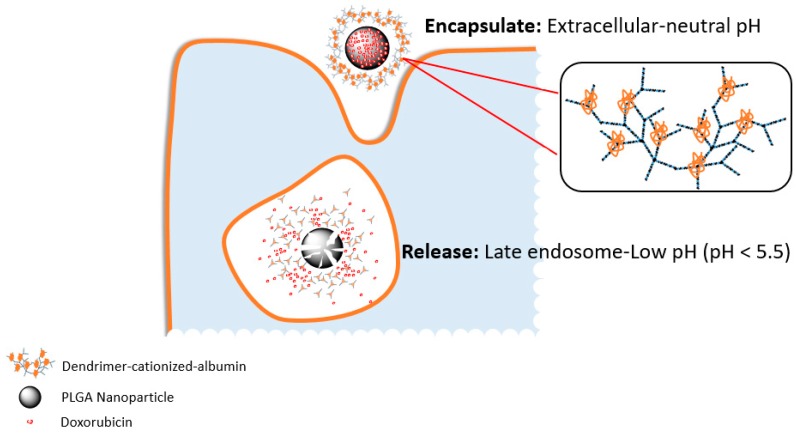
Graphic representation of the dissolution of dendrimer-cationized-albumin coated poly(lactic-co-glycolic-acid) (PLGA) nanoparticles. Doxorubicin (DOX) is released in the U-87 MG glioblastoma cells due to the pH of the acidic late endosomal compartment.

Similarly, Muniswamy et al. developed a system comprising a DOX-loaded PLGA nanoparticle core and a dendrimer-cationized-albumin outer layer (dCatAlb-pDNP). The PLGA degraded at the mid to late endosomal pH (4.5–5.5) in cancer cells and released the DOX (Figure 1). Furthermore, the system was able to penetrate the blood-brain barrier (BBB) and blood tumour barrier (BTB). Cell experiments with U-87 MG glioblastoma cells showed a 5.5 fold increase in tumour cell death due to increasing the expression levels of caspase-3 gene which mediates cellular apoptosis [22].

pH-activated release systems can also deliver cargo other than chemotherapeutics, such as nucleic acids for gene therapy techniques. Xu et al. reported an innovative pH-activated polymer (Meo-PEG-b-P(DPA-co-GMA-TEPA-C14))-based system for delivering the siRNA into tumour cells efficiently. Endosomal swelling was induced by the buffering effect of the PDPA degradation. This greatly decreased the escape time of siRNA and increased the silencing efficiency. In vitro experimental results revealed that the viability of PC3 prostate cancer cells was decreased over 5-times with the use of controlled release, and iRGD (amino acid cyclic peptide: CRGDKGPDC) polymer which enhanced the membrane penetration [20].

In addition to various kinds of polymers, many pH-responsive metallic nanomaterials have also been widely applied in tumour treatment. Titanium peroxide (TiOx) and titanium dioxide (TiO_2_) have been broadly used in biomedicine due to its biocompatibility and optical functionalities. Dai et al. reported a dual-stimuli-responsive TiOx/ DOX nanodrug system which was applied to lung cancer models. Titania nanosheets were synthesized and DOX loaded onto the surface of the sheets. Due to the exceptional pH-responsive release profile, the DOX could be preserved in the TiOx perfectly at neutral pH (releasing less than 10% over 48 h). However, the DOX could be rapidly released from the TiOx nanosheets at acidic pH, i.e., more than 50% drug release in less than 24 h. As a result, the TiOx/DOX system killed 88.45 ± 0.91% of A549 lung cancer cells with a DOX concentration of 10 μg mL^−1^ after 48h [23]. 

Iron oxide nanomaterials have been very promising pH-triggered release anti-cancer reagent carriers for a long time. Yue et al. applied iron oxide nanotubes (PMNTs) for pH-activated release of paclitaxel (PTX), which has low water solubility. The activation pH was set as 4.5, which would greatly enhance the PTX release in carcinoma cells and decrease the possibility of non-specific release in normal cells. In the stimulation test, nearly 80% of the loaded PTX was released when the pH was at 4.5, compared with less than 40% when the pH was at 7.4. At the same time, the rod-like shape of the nanotubes further accelerated the uptake rate of the system. The MTT ([3-(4,5-dimethylthiazol-2-yl)-2,5-diphenyltetrazolium bromide) assay results showed that 100 μg mL^−1^ of the PTX-PMNTs killed over 70% of the A549 cells within 24 h, illustrating the high efficiency of the system [24].

Calcium-based materials, such as calcium phosphate (CaP) [25], and calcium carbonate (CaCO_3_) [26], have also been widely applied in tumour diagnosis and therapy. Dong et al. prepared mono-dispersed CaCO_3_ nanoparticles which were coated with PEG, and carried the photosensitizer Mn^2+^-chelated chlorin e6 (Ce6(Mn)) and the chemotherapeutic DOX. While stable under physiological pH (7.4), it was demonstrated that the DOX was released in an acidic environment (pH = 5.5) due to the rapid degradation of the particles. Furthermore, in vitro experiments using 4T1 murine breast cancer cells showed that over 80% of cells were killed after incubation with 5 μg mL^−1^ DOX for 24 h. Furthermore, the release of Ce6(Mn) from the particles enhanced T1-contrast under magnetic resonance imaging. These particles are therefore suitable for photodynamic and chemotherapeutic therapy, in addition to real time monitoring. This was proven in 4T1 tumour bearing mice, in which over 25% of the T1 MRI signal came directly from the tumour, and the tumours were significantly smaller than in control groups [26].

pH-activated lipid-based nanomaterials have also showed promise in cancer treatments due to good biocompatibility and membrane penetrating functions. Choi et al. designed a nanosystem using the 1,2-distearoyl-sn-glycero-3-phosphoethanolamine-N-[biotinyl(polyethylene glycol)-2000] (DSPE-PEG) for coating MSNs with axitinib (AXT) and celastrol (CST) as cargo. In vitro drug release test results showed that the release of AXT and CST both significantly increased at pH 5.0 compared to pH 7.4. The results from the systems revealed that the lipid outer layer significantly increased the efficacy of the mitochondrial-based cell apoptosis. When the system was applied to SCC7 carcinoma tumour bearing mice models tumour inhibition was over 64% compared with the control groups [27].

#### 1.1.2. Enzymatic Activation of Nanoparticles 

Enzymes in the body catalyze more than 5,000 biochemical processes [28]. It has been shown, however, that enzyme expression levels in tumour tissues can be significantly higher than those in normal tissues [29,30,31]. This raises the possibility for enzymes to activate nanoparticle systems specifically at the tumour site [29,31]. A number of cellular enzymes have been used in enzymatic activation systems, including cathepsins [32,33], matrix metallo-proteinases (MMP) [33,34], hyaluronidase (HAase) [35], glycosyl hydrolases [36], NAD(P)H-quinone oxidoreductase-1 (NQO1) [30,37], protein tyrosine kinase-7 (PTK-7) [38], and telomerase [29,39].

##### – Cathepsins

The expression rate of cathepsins has been shown to be related to the invasiveness and metastasis of various types of cancers, such as breast cancer, ovarian cancer and pancreatic cancer [40,41]. Polymers such as poly-L-lysine hydrobromide (PLL) can be specifically degraded by lysosomal cathepsin B. As such, Villar-Alvarez et al. reported a particle coating with PLL and incorporated gold nanorods and the chemotherapeutic DOX. The assembled system could decrease the viability of HeLa cells and MDA-MB-231 cells to around 20% when incubated with 2.5^10^ NPs/mL; the same concentration of free DOX killed less than 50% of the cancer cells [42]. Similarly, Yildiz et al. described two protease degradable amphiphilic copolymers, poly(lactic-co-glycolic acid)-b-poly-L-lysine (PLGA-PLL) and poly(lactic acid)-b-poly(ethylene glycol) (PLA-PEG), which could react with cathepsins. The copolymers were conjugated to a NIR fluorophore and used to image tumour tissue non-invasively at the same time as carrying a cytotoxic drug to tumour tissues. The drug was released after the copolymer was biodegraded by the high concentration of lysosomal cathepsins in cancer cells [43]. 

##### – Matrix Metalloproteinases

Matrix metalloproteinases (MMP) play critical roles in cancer development and progression, including invasion and metastasis. There are 23 types of MMPs in humans, which are grouped and numbered based on their specificity to certain substrates and cellular locations. Collagens, gelatin and extracellular matrix proteins are the main substrates for the MMP family [29,33,34,44,45]. Both MMP-2 and MMP-14 have shown potential as activating biomarkers for cancer treatment nanosystems [29]. The MMP-9 has been shown to dissolve a gelatin/ polyvinylpyrrolidone (PVP) coating layer, which could lead to responsive release in tumours. This has been incorporated into a proof-of-concept system in which mesoporous silica nanoparticles were loaded with dye molecules and the surface coated with PVP [34]. The dye molecules are only released after enzymatic degradation of the PVP layer. The signal was examined by multispectral optoacoustic tomography (MSOT) and shown to be 10 times greater for the MMP-9 treated group compared with the non-treated control group.

##### – Glycosyl Hydrolases

Glycosyl hydrolases are a class of intracellular enzymes which catalyze the hydrolysis of glycosidic bonds in complex sugars [46], but which have also been investigated as an activation agent for controlled release. An elegant system was proposed by Dzamukova et al. in which a dextrin cap was applied which could be degraded by glycosyl hydrolases. A clay tube-like nanoformulation was used to encapsulate a cytotoxic drug with dextrin functioning as the tube-end stopper. Consequently, in the presence of high levels of glycosyl hydrolases in cancerous tissue the drug could be released after decomposition of the dextrin [36]. 

##### – Protein Tyrosine Kinases

Protein tyrosine kinase-like 7 (PTK-7) is one of a subgroup of kinases which lack catalytic activity but retain roles in signal transduction. They have been shown to have significantly higher expression levels in some specific cancer types, such as human T-cell acute lymphocytic leukaemia (CEM) and oesophageal squamous cell carcinoma (ESCC). Although the role of PTK-7 hasn’t been fully discovered, it is recognized as being related with the cancer progression [47]. In 2014, Huang et al. reported an aptamer-lipid-PLGA hybrid nanoparticle which could co-delivery DOX and paclitaxel (PTX). In this system, specifically selected aptamers interact with PTK-7 expressed on the membrane of tumour cells. Upon interaction of the aptamer with the kinase, the aptamer structure changes, and the DOX which was bound in the hairpin structure of the aptamer is released [38]. 

##### – Nicotinamide Adenine Dinucleotide Phosphate (NADPH) Dehydrogenases

While there are many members of the NADPH dehydrogenase family, the expression level of NAD(P)H: quinone oxidoreductase-1 (NQO1) is approximately 12–50 fold higher in tumour cells [30,37]. Based on this, Shin et al. reported an innovative theranostic nanoprobe called Prodrug 1. Prodrug 1 is decomposed by the action of NQO1 into the toxic compound 7-ethyl-10-hydroxycamptothecin (SN-38) which is a well-known chemotherapy drug [30]. 

##### – Telomerases

**Figure 2:**
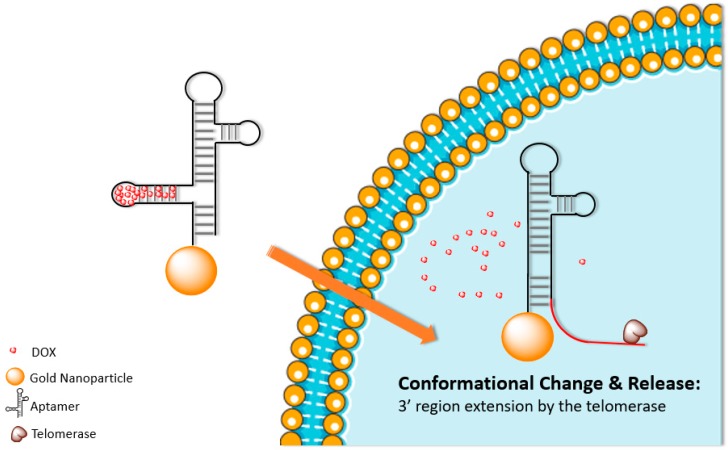
Graphic representation of telomerase extending the 3’end aptamer which leads to the conformation change of the aptamer and release of the DOX inside tumour cells.

Very recently, Shi et al. developed a unique telomerase activity-triggered DOX release theranostic nanoprobe [39]. Telomerase activity is much higher in cancerous cells than in normal cells. Therefore, a unique hairpin DNA shell conformation was designed that included a 3’ telomerase primer region, which would be elongated by the existing telomerase (Figure 2). As the telomerase activity in cancerous cells is much higher than the normal cells, the elongation of the 3’ region would cause the deconstruction of the aptamer structure and lead to the release of the anticancer DOX and fluorescent 5-carboxyfluorescein (FAM) label which was bundled inside the aptamer. Using MTT assays the nanoprobe was shown to decrease the growth of HeLa cells by over 50%, compared with almost no decrease in the viability of control L-02 cells [39].

##### – Dual Enzyme Activation

In addition to systems which use a single specific enzyme, dual enzyme activatable systems have also been developed to increase specificity. Han et al. reported a gemcitabine-poly(ethylene glycol) coated CdSe/ZnS quantum dots (QDs) nanosystem which interacts with both MMP-9 and cathepsin B. As a result, the dual enzymatic activatable system could greatly increase the efficiency and accuracy of treatment agent delivery and extend circulation time within the body [33].

##### – Enzyme Loaded Nanoparticles

In addition to making use of those enzymes already present within cells, it is also possible to design systems which carry enzymes into the body. An example of this was developed by Yang et al. using an organic silica-based nanosystem which carries the hypoxia-activated prodrug (AQ4N) and glucose oxidase (GOx). High glutathione (GSH) levels within the tumour tissues enables the degradation of the organic silica framework and the release of GOx and AQ4N. The GOx catalyses glucose oxidation leading to an hypoxic environment within the tissues, which then enables the non-toxic AQ4N to transform into the cytotoxic AQ4 [48]. Using a similar system, Lian et al. reported unique metal-organic frameworks (MOFs) for enzyme encapsulation. The MOFs could provide robust protection for enzymes to ensure that catalytic activities were maintained. In this study, the nontoxic pro-drug Paracetamol (APAP) could be efficiently transformed into the toxic 4-acetamide-o-ben-zoquinore (AOBQ) with the appearance of transported tyrosinases [49].

#### 1.1.3. Concentration Dependent Activation 

A variety of molecules are found at different concentrations in healthy cells compared to cancerous cells due to abnormal gene expression and metabolism. These differences could be exploited for activation systems and can be separated into: (1) membrane proteins which are normally related with the over-expression of a gene and could enhance the penetration of nanosystems into the cell via internalization functionalities; and (2) soluble molecules which are normally related with the changed metabolism and could be used to activate the nanosystems directly.

##### – Membrane Proteins 

There is an extreme demand for iron in cancerous cells due to the high rate of proliferation. This has been demonstrated in cancers such as melanoma [50], carcinoma [51], and glioblastomas [52] amongst others. Transferrins are iron-binding glycoproteins which control the level of free iron in biological fluids. Iron ions can be transported into cells by the binding of transferrin to transferrin receptors (TfRs) on the surface of cells [53]. Transferrin receptors can therefore be used for tumour targeting due to the requirements for high levels of iron in the cancer cells [54]. Targeting using TfR was demonstrated by Zhang et al. for the deep delivery of nanosonosensitizers into tumours. The nanosonosensitizers were composed of protoporphyrin IX (PpIX) conjugated human holo-transferrin. Unlike the passive tumour diffusion of NPs relied upon by many systems, this construct makes use of the TfR-mediated endocytic pathway for tumour penetration. Once inside the tumour cells, PpIX can be activated by ultrasound and generate reactive oxygen species (ROS). This was demonstrated in HeLa cells and multicellular tumour spheroids (MCTS). Cell apoptosis in the treatment group (NPs+ ultrasound) was significantly higher than the control group and ultrasound only group (~60% vs. ~40%). Furthermore, the penetration enhancement was confirmed by the detection of PpIX fluorescence in the whole transverse surface of MCTS even at a depth of 80 µm [55].

Epidermal growth factor receptor (EGFR) is also known to be overexpressed on tumour cell membranes. It has been widely recognized that EGFR overexpression is related to treatment resistance [56,57], and poor prognosis [58]. However, the receptor can be activated by the binding of ligands which can be incorporated onto nanosystems and lead to the internalization of the receptor and ligand-NP [59]. Antibody-drug-conjugates (ADCs) have been trialled for clinical tumour treatment [60], however, the amount of anti-cancer drug which can be carried is limited, and may lead to the failure of therapy or treatment resistance [61]. To address this, Roncato et al. reported a unique anti-EGFR antibody (cetuximab) conjugated avidin-nucleic-acid-nano-assemblies (ANANAS) which contained around 50 avidin and 1200 biotin-binding sites (BBS) for further functionalization. The construct was conjugated with cetuximab and DOX, and the cetux10-ANANAS-doxo system was tested against immortalized breast cancer cells. The nanosystem which contained 10 µM of DOX killed over 70% of the EGFR over-expressing MDA-MB-231 cells in 72 h. This is in comparison with the cetux-doxo ADC with same concentration of DOX which only resulted in around 40% cell death. The nanosystem was also tested against MDA-MB-231 tumours grown in the flank of NOD/SCID (nonobese diabetic/severe combined immunodeficiency) mice. The nanosystem showed significantly greater suppression of the tumour growth than the control group, the corresponding ADC, or even a ten times higher concentration of pure DOX [62].

Prostate-specific membrane antigen (PSMA) was shown many years ago to be overexpressed in all types of prostate adenocarcinoma together with some kinds of breast, lung and colon carcinomas [63]. The PSMA has the potential to be applied not only as a target for tumour therapy, but also to enhance the uptake rate of nano-vesicles carrying therapeutic reagents through its internalization motif [64,65]. Mangadlao et al. described a system in which a nanoparticle is conjugated with the PSMA receptor binding ligand PSMA-1 and the fluorescent photodynamic therapy drug Pc4. The nanosystem is designed to first accumulate at the tumour site through the Enhanced Permeation and Retention (EPR) effect, and then for the PSMA-1 ligand to bind to the cancer cell membrane, and be internalized, followed by laser activation of the Pc4 molecule. Cells expressing PSMA (PC3pip) were incubated with the NPs treating and exposed to a laser (1.0 J). After 24 h, almost all of the cells were killed. As a control, PSMA non-expressing PC3flu cells were treated similarly, and shown to have a 35% survival rate [66]. 

**Figure 3:**
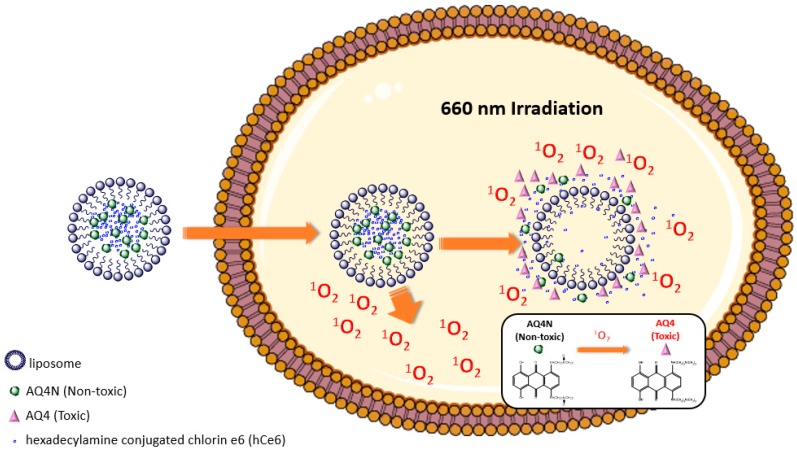
The singlet oxygen concentration was further enhanced by hCe6- hypoxia-activated prodrug (AQ4N)-liposome nanosystem irradiated by 660 nm laser and non-toxic AQ4N was transformed to toxic AQ4 by high concentration of singlet oxygen.

Many proteins in cancer cells are differentially expressed due to the hypoxic conditions. This arises because of low oxygenation as a result of abnormal and inadequate blood vessels, especially in the centre, of solid tumours. Hypoxic cells are resistant to many chemotherapy and radiotherapy agents, and often leads to a poor prognosis [67]. However, as a common functionality of tumours, hypoxia could be exploited for new activatable systems. Feng et al. developed a hypoxia-activated theranostic nanosystem based on a multipurpose liposome. By the hydrophobic-hydrophilic interaction, the hydrophilic prodrug AQ4N was conjugated to the hydrophobic photosensitizer hexadecylamine and chlorin e6 (hCe6) and liposomes (Figure 3). Under 660 nm light irradiation, the AQ4N-hCe6-liposome system could efficiently generate large amounts of singlet oxygen leading to a wider hypoxic environment inside the tumour. The AQ4N, which is non-toxic in a normal environment, was activated by the hypoxic environment and transformed to the toxic AQ4 form which could kill the tumour cells. The therapeutic effects of the AQ4N-hCe6-liposome system were then further evaluated in female Balb/c mice bearing with 4T1 breast tumours. As a result, with the corresponding dosage of hCe6 and AQ4N at 10 and 5 mg kg^−1^, the relative tumour volume was almost unchanged for 14 days with 1 h of light irradiation every day (2 mW cm^−2^). In contrast, the relative tumour volume increased around seven times in the saline injected control groups which illustrated the therapeutic effects of the hCe6-AQ4N-liposome nanosystem [68]. 

##### – Soluble Molecules

Intracellular glutathione (GSH) is much more concentrated inside cells (2–10 mM) than extracellularly (2–10 µM) [4]. Furthermore, the intracellular GSH concentration of cancerous cells is normally 1.7–7 fold higher than in normal cells [4]. Yang et al. reported a cancer cell specific degradable dendritic mesoporous organosilica nanoparticle (DDMON) utilising the differences in GSH concentration for selective release. The release mechanism was based on the disulfide bonds (-S-S-) incorporation in the DDMONs. The high concentration of GSH in the cytoplasm of cancer cells would lead to the disintegration of the NPs structure by the reduction of the disulphide bonds, and the release of encapsulated therapeutic agents. The DDMONs which were generated carried the cancer therapeutic PEI/RNase (Polyethylenimine/Ribonuclease A) A-Aco-FITC (cis-aconitic acid- Fluorescein isothiocyanate) complex. Incubation of the complex in melanoma (B16F0) cell lines, resulted in significantly higher cell kill compared to the same amount of the loaded non-biodegradable dendritic mesoporous organosilica nanoparticles (DMONs). As a control the constructs were tested in normal HEK293t cells, and conversely viability was higher after treatment with DDMONs than DMONs. This confirmed the selectivity of the system based on the GSH concentration, thereby providing protection for healthy cells [69].

## 2. Extrinsic Activation 

### 2.1. Ultrasound Activation

Ultrasound has been used in medicine since the 1930s for both diagnostic and therapeutic purposes, due to its non-invasive, non-ionising and generally safe nature. Ultrasound irradiation works by an identical mechanism to soundwaves; positive and negative pressures produced by waves cause both thermal and non-thermal effects through mechanical friction [70]. 

#### 2.1.1. Sonoporation 

When ultrasound is applied to cells it causes an increase in their porosity; an effect known as sonoporation. This effect can be combined with microbubbles to enhance the effect further. Although hypotheses about the exact mechanism vary, it is believed that increased uptake is a result of stable or inertial cavitation of the microbubbles [71]. During stable cavitation a microbubble oscillates under the alternating pressure of the applied ultrasound and causes streaming of fluid around the bubble. This causes shearing and fracture of the cell membrane and hence increased porosity. In this mechanism the microbubble remains intact even after the interaction. Alternatively, inertial cavitation can be induced, in which the bubble collapses under the alternating pressure and produces microjets and shockwaves that lead to pore formation. These induced pores can be either transient or permanent; permanent porosity will eventually lead to cell death, but cell death in this manner is generally uncontrollable and undesired in remote activation of nanoparticles [72]. 

#### 2.1.2. Gas Filled Microbubbles

Microbubbles for medical use typically comprise a perfluoro carbon gaseous interior and a polymer or protein biocompatible shell. Gas-filled microbubbles can be used for the delivery of nanoparticles by chemical attachment or encapsulation on or within the microbubble, followed by ultrasound irradiation [71,73]. Independent studies have shown a range of increased nanoparticle uptake of between 5–57 and 60–600 fold when administering the nanoparticles using microbubble attachment. In addition, the effective penetration depth of nanoparticles in tissue can be increased by hundreds of micrometres [40,74,75,76]. 

Successful delivery of nanoparticles bonded to the surface of microbubbles relies on the localized destruction of the microbubbles within the tumour blood vessel and subsequent distribution of the bound nanoparticles to the surrounding tumour tissue [74,76,77,78]. This technique has been used for the delivery of compounds such as doxorubicin. Polymer microbubbles have also been formulated which contain doxorubicin. In the latter, the drug is encapsulated using an emulsion method, and when the shell is ruptured by ultrasound irradiation the drugs are released into the tissue [39,79]. While it was successful, only a low loading capacity of doxorubicin in the bubbles was achieved and so the system was adapted to encapsulate the very potent microtubule stabilizing drug paclitaxel [42,79]. 

A drawback of adhering nanoparticles to microbubbles is the vast reduction of circulation time in vivo. In addition, the microbubbles often collapse upon ultrasound irradiation, and hence cavitation times are very short. Recently, however, nano-cones and nano-cups have been designed that increase and sustain cavitation times four-fold, and retain the long circulation times nanoparticles possess. Within the single cavity of the nano-cones and nano-cups, a nanobubble is formed and stabilised. Upon ultrasound irradiation the particles are propelled into surrounding tissue and uptake is again aided by cavitation and subsequent sonoporation. Gold particles of this nature have been used to deliver IgG mouse antibody in vivo, increasing delivery distances from blood vessels to hundreds of micrometres [40,75]. mRNA-lipoplex loaded microbubbles have also been shown to increase transfection of dendritic cells from negligible amounts to 50%, when ultrasound is used as an activating stimulus [73].

#### 2.1.3. Combinatorial Ultrasound

An innovative combinatorial mechanism has been developed incorporating the anti-proliferative effect of sonodynamic therapy with a pH-activated chemotherapeutic action [80]. TiO_2_ particles doped with Gd served as a sonosensitiser and a nanocarrier for the delivery of Doxorubicin [81]. The nanoparticles accumulated within tumour tissue via the EPR effect and a pH responsive polyethylenimine coating released the drug payload. Once inside the cell, protonation of the amines in the coating results in an influx of counter-ions and a lowering of the osmotic potential. Osmotic swelling results and bursts the polymer shell releasing the polymer-doxorubicin complexes into the cell. Subsequent ultrasound irradiation at the tumour site then initiated ROS production which was catalysed by the Gd doped surface of the particles. This results in a two-pronged approach which can selectively initiate cell death only within the cancerous tissue. 

Calcium carbonate nanoparticles have also been used in conjunction with ultrasound for enhanced activity. pH sensitive calcium carbonate was encapsulated within a poly(D-L-lactide-co-glycolide) coating. The surface was modified with Rabies virus glycoprotein which was used to target the particles to neuroblastoma cells in tumour bearing mouse models. In the low pH environment of tumour tissue, the calcium carbonate dissolves to form carbon dioxide gas; this can assist ultrasound imaging. In addition to increasing imaging capability, the nanoparticles and ultrasound in combination slowed the growth of the tumours (to a much greater extent than either ultrasound or particles alone) [82].

Activated imaging has also been achieved with lipid (DPPE-PEG)-coated, perfluorocarbon-filled particles formed as nanoscale lipid droplets. Upon ultrasound stimulation the droplets are vaporised into gaseous microbubbles for imaging and therapy. Fluorescent dyes in the shell of the lipid layer also enable microscope imaging and tracking the phase change of the molecules [83]. Folate receptors were also added to the construct to target MDA-MB-231 and MCF7 breast cancer cell lines, and the phase change after activation showed excellent uptake of the contrast agents, and good imaging capabilities.

#### 2.1.4. Piezoelectric Nanomaterials 

**Figure 4:**
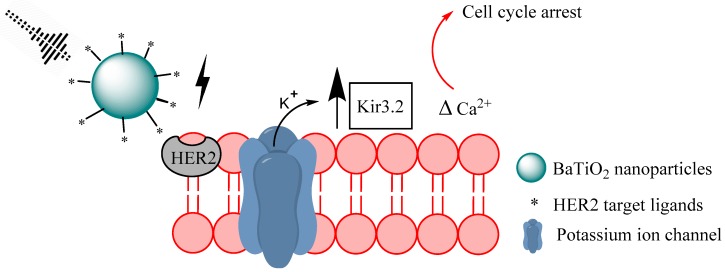
Graphic representation of ultrasound stimulated piezoelectric stimulation of breast cancer cells. Upon ultrasound induced deformation, barium titanate nanoparticles create a potential difference that interferes with Ca^2+^ homeostasis and upregulates the Kir3.2 gene.

The material composition of the nanoparticles can be a crucial aspect in the design of an ultrasound release system. Piezoelectric nanomaterials are known to produce an electric potential difference on their surface when they undergo mechanical deformations. Upon ultrasound radiation, a piezoelectric semiconducting coupling process converts mechanical energy into electricity [84]. This potential difference can disrupt cell division by affecting K^+^ channels and by affecting the organization of mitotic spindles during mitosis. Since low-intensity electromagnetic fields are able to reduce cancer cell proliferation by inducing intracellular Ca^2+^ increments, by interfering with Ca^2+^ homeostasis [85]. Barium titanate piezoelectric nanoparticles have been further functionalised with the anti-HER2 antibody (Figure 4), and have been reported to successfully inhibit proliferation of breast cancer cells under ultrasound stimulation [86].

### 2.2. Magnetic Field Activation 

Magnetic nanoparticles show potential in oncology for their use in induced magnetic hyperthermia, localized drug delivery by external magnetic fields, and also as contrast agents in magnetic resonance imaging (MRI). The most frequently used materials are magnetite (Fe_3_O_4_) and maghemite (γ-Fe_2_O_3_).

Magnetic nanoparticles are able to convert electromagnetic energy into heat. This is typically accomplished by the hysteresis loop mechanism in alternating magnetic fields (AMFs) due to either Néel relaxation, Brownian motion, or possibly particle-particle interaction in super-paramagnetic nanoparticles at frequencies between 100–300 kHz [87]. The efficiency of heat generation is dependent upon magnetic field strength and frequency, nanoparticle size, concentration, and solution viscosity [88]. Alternating magnetic fields have long been used to induce hyperthermia for a variety of medical treatments. The main advantage in using an alternating magnetic field as a stimulus lies in its non-ionising and specific nature. 

#### 2.2.1. Magnetically Induced Hyperthermia

Since the development of hyperthermia treatment in the 1970s this method has been the focus of intense research both as a sole therapeutic method, and in combination with other treatments. When used in combination with radiotherapy, hyperthermia treatment has shown excellent results in a variety of cell lines [89]. With the discovery of the very high specific absorption rate of iron oxide nanoparticles (IONs), subsequent hyperthermia research greatly increased in effectiveness and specificity, leading to the first clinical trials in 2009 [90]. The inductive heating effect of IONs is caused when an alternating magnetic field (AMF) constantly flips the magnetic orientation of the superparamagnetic magnetite particles. Superparamagnetism occurs when a ferro/ferrimagnetic material becomes suitably small, that is, ~10 nm, and behaves as a single spin system. Rapid alternation of the magnetic orientation may then generate heat after internalization, and cell death can occur when temperatures reach approximately 42 °C [90,91]. The inductive heating effect from magnetic field stimulation can be used in conjunction with a chemotherapy system in which release is triggered by a temperature change. 

Magforce has developed the NanoTherm^TM^ therapy for treating brain tumours. The iron oxide particles are 14 nm in diameter with an aminosilane coating, and the ferrofluid is used at a concentration of 17 quadrillion particles per millilitre. NanoTherm^TM^ is inserted into the tumour and the coating causes the particles to remain in situ, permitting multiple treatment cycles. Their proprietary Nanoactivator^TM^ creates an alternating magnetic field such that the magnetic field alternates around 100,000 times per second, and the rapid change in the nanoparticles magnetic orientation activates the particles resulting in heat. Using such an approach the tissue can reach temperatures of up to 80 °C, directly destroying cancer cells. The technology was tested in clinical trials for glioblastoma and used in conjunction with radiotherapy. Conventional treatment showed a median survival time of 6.2 months, compared to 13.4 months for the 59 patients using NanoTherm^TM^; more than double. The technology is being trialled in prostate cancer patients; the first patients were enrolled in late 2018, and is expected to take 12–15 months to complete. There are however, two drawbacks to this technology, firstly all metal must be removed from within 40 cm (e.g., dental work) and secondly MRI can never be used again, including for diagnosis of tumour progression. 

Therapies involving the inductive heating of gold nanoparticles have been a topic of hot debate for some time. Gold has been shown empirically to demonstrate inductive heating upon application of an AMF. Theoretically, however, gold does not possess an electronic configuration that would allow such a process, and it has been suggested that nano-surface and quantum electronic effects were the cause [92,93]. In 2017 it was concluded that the temperature change observed by investigators was a result of the movement of the ion layer surrounding gold particles in a colloid, and the conjugation of proteins in solution with the surface of the gold particles [94]. Despite the controversy over mechanism of heating, the activation of gold NPs by AMF has proven effective. Antibody-conjugated gold nanoparticles have been shown to actively accumulate in liver cancer cell lines (Panc-1, Hep3B, and SNU449) and cause hyperthermia and subsequent cell death under AMF [93]. A smart biosensor consisting of a gold nanoparticle core, a DNA ‘melting stem’ coded for the recognition of tyrosine hydroxylase and a one reporter fluorochrome allows fluorescence imaging under a 3 GHz AMF [92]. Although there is not the range and depth of research into gold nanoparticles activated by AMF compared to IONs, this is a promising area and retains potential for further development. 

#### 2.2.2. Magnetically Induced Localized Drug Release

Liposomes and micelles carrying IONs have been used as a means of encapsulating and delivering therapeutic drugs to tumour sites. The shells are commonly based on a thermosensitive polymer such as poly-N(isopropylacrylamide) or poly(vinylcaprolactame). The liposomes, while varying in construct and payload, are typically activated by two methods; the first involves the inductive heating of the internal IONs by an AMF and a subsequent structure change of the thermosensitive polymer making up the liposome. The structure change releases the entrapped drugs and can be reversible or irreversible depending on the polymers used. The second method involves the application of a permanent magnetic field, causing the IONs to drag and squeeze the liposomes to a point at which they burst and the payload is released [43,95,96,97,98]. Nanocapsules designed in this manner by an emulsion approach have enabled the simultaneous activated delivery of both hydrophobic and hydrophilic drug molecules contained within the same vesicle. Utilising block polystyrene acrylic acid copolymers, compounds such as FITC-DNA, fluorophores and pyrene are contained within their phases until activated [98]. When activated in this manner drug delivery can have a high specificity and payload efficiency. However, it is not a perfect system and the size and composition of the micelles or liposomes can vary hugely during synthesis and often result in unwanted effects on the penetration of treatment into tumour tissue, thus hindering the efficiency of delivery in vivo. 

Improved tumour uptake and localization of compounds can be achieved using magnetite-based nanoparticles. Fe_3_O_4_ core nanoparticles with a poly(N-(1-1-butyric acid)) aniline shell have been shown to improve the delivery of carmustine from within the polymer, shown active targeting in directed magnetic fields, and can simultaneously serve as a contrasting agent for MRI imaging. In addition to this, the nanocarrier improved drug half-life from 18 to 62 h, and improved survival rates by 20% in mice models compared to administration of the drug alone [99,100]. Another combinational treatment method uses cationic, biocompatible peptide dendrimers loaded with doxorubicin and grafted onto the surface of ION’s. An applied AMF induces hyperthermia in cells and promotes the release of the chemotherapeutic drug giving a best of both worlds treatment. These particles showed high rates of cell death in HeLa, PC-3, MCF-7, and KB cell lines [101]. 

A proof-of-principle system for stimulating insulin release has been developed using a modified temperature sensitive TRPV1 channel in conjunction with antibody coated Iron Oxide Nanoparticles (IONs). The IONs were coated with anti-histidine (His) which bound to an extracellular His epitope tag on the modified TRPV1 channel. Application of the AMF inductively heated the IONs such that the temperature sensitive TRPV1 channel generated a calcium current. This in turn activated the Ca^2+^ sensitive promoter of the human insulin reporter gene [102]. Not only does this method utilise the stimulus responsive activation of nanoparticles, but it demonstrates the activation of cells using nanodevices. The system shows potential for use in stimulating tumour cells to self-destruct. 

Advances in thermosensitive polymers and the optimisation of inductive heating methods has resulted in an increase in research into trapping drugs and IONs within a polymer matrix for delivery to tumour sites. A combination particle comprising a poly(ethylene oxide)-poly(propylene oxide)-poly(ethylene oxide) triblock copolymer matrix containing polyvinyl alcohol shell, ION and drugs such as ethosuximide has shown incredibly tuneable drug release upon stimulation. The reversible collapse of the copolymer and the swelling of the PVA core upon temperature rise provides an outstanding mechanism of drug release [103]. In contrast, nanocomposites of poly-N(isopropylacrylamide) matrices and ION showed the suppression of drug release upon inductive heating. Owing to the collapse of the polymer and the closure of pores in the matrix, this provides a means of turning off drug delivery once the desired therapeutic effect has been achieved, reducing the potential for off-target toxicity [104].

Core shell structured nanoparticles have been designed that provide an almost zero release profile of drugs unless activated by an AMF. A contemporary example consists of a PVA-ION-hydrophobic drug core surrounded by a thin layer of silica, which can help regulate the release pattern. A burst release occurs when the inductive heating effect of the AMF causes disintegration of the inner core [105]. In a mirrored manner to this, a core shell system with a drug containing silica core surrounded by an iron oxide crystal shell has been created. Again considerable release is only seen after an external field is applied [106]. Utilising functionalised mesoporous silica nanoparticles ca.100nm in diameter, methylene blue for fluorescence imaging and ION are entrapped within the pores of the silica particles and capped with a lipid bi-layer. Upon AMF stimulation and subsequent ION inductive heating the bi-layer is broken and the payload of the pores released [107]. This innovative design has the potential to have even greater effect if the payload is optimised to carry therapeutic drugs and the bilayer surface engineered with receptor targeting ligands.

#### 2.2.3. Smart Stimulus Systems

**Figure 5:**
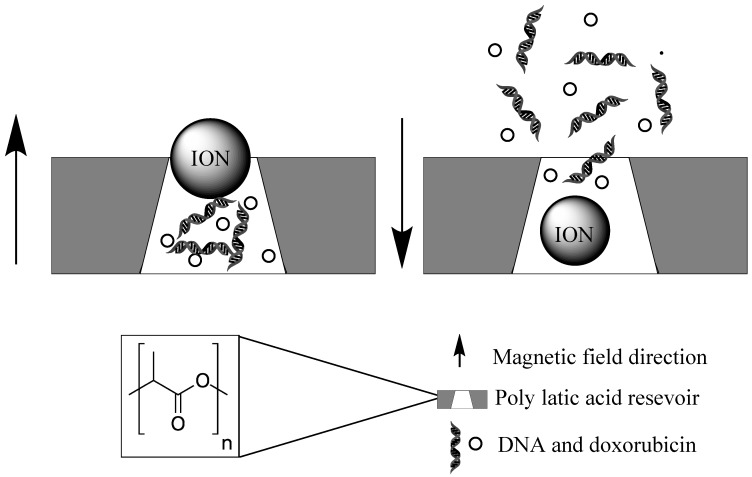
On-off tuneable release mechanism of chemotherapy molecules and gene therapy for DNA. The pores of the polymer reservoir are blocked by magnetic field susceptible ION nanoparticles.

A rather ingenious nanodevice was developed using magnetite particles as a pore blocking instrument to selectively release vitamin B12 and DNA, in a pulsed or burst manner. While switched off the magnetite particles act as a blocking device, preventing the release of drugs from within the reservoir. The multi-reservoir device is made from a biodegradable polymer (poly lactic acid) and upon application of an external magnetic field, the magnetic particles are dragged to the bottom of the wells and the drugs released (Figure 5). Once the desired release concentration has been achieved the magnetic field direction can be flipped and the pores closed [108]. A similar gatekeeping release system has been demonstrated with zinc doped iron oxide nanocrystals (ZnNCs) within a mesoporous silica shell. The ZnNCs are synthetically positioned at the core of the MSNPs. A molecular thread is then bound to surface in readiness to bind to a capping agent. Doxorubicin is then loaded into the pores of the silica and the capping agent, cucurbit[6]uril, bound to the thread. Upon inductive heating of the interstitial ION, the electrostatic bonds between the valve and the silica surface are broken and the drug released [109].

A method of triggering ultrasound activated sonodynamic therapy by radio-wave stimulation has been created. This system used KF doped BaTiO_3_ dielectric nanoparticles contained within a poly(N-isopropylacrylamide) matrix which enables the regulation of sound waves by electromagnetic irradiation [110]. When combined with other ultrasound stimulated methods (also mentioned in this review) these methods could indeed overcome some of the problems with ultrasound activation, such as activation in the lungs or ribs. 

Magnetofection can also be used to enhance gene transfection efficiency via magnetic field-enforced cellular transport processes. Magnetite nanocrystal clusters, cross-linked with polyethyleneimine have been developed which can magnetically trigger intracellular delivery of small interfering RNA [111]. This has potential in gene therapy applications. 

### 2.3. Light Activation and Photodynamic Therapy 

Photodynamic therapy (PDT) has seen rapid progress since the first modern use in 1975 [112]. Unlike chemotherapy and radiotherapy the systematic toxicity is very low. Nanotechnology has found a place in PDT treatment to improve the efficiency, localisation and penetration depth of conventional PDT. Upconversion nanoparticles (UCN) have been a key contributor to advances in PDT. Photon upconversion involves the sequential absorption of two or more photons which results in the emission of light at a shorter wavelength than the excitation wavelength. The UCNs are therefore commonly excited with visible light wavelength to generate ultraviolet light. Alternatively, near infrared light can be used as the excitation source due to its minimally damaging tissue effects, and high penetration depths. Typically, the upconversion material comprises crystals of NaYF_4_ doped with either Yb/Er or Yb/Tm; the choice of activator dopant is influenced by comparative energy levels [113]. 

Using the principles of UCN and traditional PDT, many methods of visible or near infrared light stimulated chemotherapy have been developed. Using light as a stimulus mechanism has allowed localized release, reduced incident light effects and synergistic treatments to be developed. The illumination of photosensitisers within the patient generating ROS and subsequent tumour destruction, provides a means of effective, non-invasive treatment. 

This section will also focus on light activated chemotherapy as well as advancements in PDT.

#### 2.3.1. Near Infrared Light Activation

Near infrared radiation (NIR) specifically 700–1200 nm, has been used as a stimulus for PDT since the inception of upconverting nanomaterials [114]. Visible light can only penetrate <1 cm into body tissue and intense prolonged exposure will cause burns and lesions [112,115]. NIR light increases penetration depth by several fold and reduces the possibility of damage to healthy tissue [116,117,118,119]. Gold nano clusters have been designed that in response to NIR activation, can image, assist gene delivery, and enable PDT in HeLa cells. Gold nanoclusters conjugated to TAT-peptide were shown to actively accumulate in the nucleus and enable red fluorescence imaging in vitro and in vivo. In addition to imaging, the peptide catalyses singlet oxygen production and serves as a DNA delivery medium, giving ultrahigh uptake (90%) and transfection (80%) [119]. 

Imaging has also been performed with PLA-PEG-FA polymers encapsulating a NIR sensitive carbazole substituted boron-dipyrromethene photosensitiser. These small, biocompatible, organic nanoparticles were used with an ultralow power lamp (670–800 nm) and tumour volume reduction was on average around 80% in mice bearing subcutaneous 4T1 tumour xenograft [118]. The photosensitizer TiO_2_ can also be adapted to respond to NIR light. The addition of an NaYF_4_:Tm upconverting shell permits the activation by NIR light. After coating with PEG, particles were shown to be hydrophilic, biocompatible and result in >70% cell death of oral squamous cell carcinoma (OSCC) cells after NIR exposure [116].

Controlled drug release can also be achieved using NIR light. A unique approach using black phosphorous nano sheets and a low melting point agarose hydrogel matrix has shown fully controllable drug release. Black phosphorous nano sheets show a very high photothermal conversion under NIR, therefore after activation in vivo the hydrogel degrades and releases encapsulated therapeutic molecules. A key feature of this system is the control over the rate of drug release (light intensity and exposure time) and the biocompatibility of the breakdown products after degradation [120]. The NIR mediated release of doxorubicin has also been shown using gold nanorods coated with a mesoporous silica shell. Pores in the silica were capped with the phase changing molecule 1-tetradecanol, and upon NIR heating of the gold nanorods the chemical caps change phase and open the pores, releasing the drug. In addition to doxorubicin release, under intense NIR, the nanorods were also able to induce hyperthermia in the tumours. The nanoparticles were tested in KB cells (a sub-line of the cervical cancer HeLa cell line) and showed massive cell death in response to NIR exposure [121]. A further example of a NIR responsive with a mesoporous silica shell incorporates a NaYF_4_:Yb_0.4_/Tm_0.02_@NaGdF4:Yb_0.1_@NaNdF_4_:Yb_0.1_ core. Doxorubicin is retained within the porous shell by photocleavable platinum (IV) complexes. Upon NIR exposure and upconversion by the core to UV light photons, the platinum (IV) complex breaks down into a chemotherapeutic platinum (II) complex and simultaneously opens the pores and releases doxorubicin [117]. Chemotherapy and PDT were also combined in a system using nanoparticles with a polymer core containing a payload of cisplatin surrounded by an ordered asymmetrical shell of cholesterol and PEG, as well as the photosensitizer pyrolipid. Upon 600 nm irradiation the lipid layer is ruptured and the therapeutic molecule released in addition to ROS generated by the photosensitizer [122]. However, PDT can sometimes result in hypoxia. To tackle this, a smart system was designed to incorporate ROS-generating and hypoxia-sensitive 2-nitroimidazole-grafted conjugated polymer (CP-NI), polyvinyl alcohol-based surface coatings, and encapsulated doxorubicin hydrochloride. When exposed to NIR the polymer shell will generate ROS, this will cause cell death or may induce hypoxia, and in turn this causes the rupture of the nanoparticle and releases the encapsulated doxorubicin [123]. Targeting has also been extensively incorporated into nanoparticle systems to improve selectivity. One such example uses a β-NaGdF_4_:Yb,Tm@NaGdF core to produce ROS in conjunction with the photosensitizer PC70, and a PEG coating for biocompatibility, which were also chain capped with folic acid. The nanoparticles were tested in HeLa-luc cells both in vitro, and in vivo within female mice; in both cases tumours were completely destroyed and negligible side effects were observed [124]. 

#### 2.3.2. Visible Light Activation

Whilst the rare earth elements are commonly used due to their roles in upconversion systems, other metals have been used in visible light activated systems. Zinc phthalocyanine is a known photosensitizer, and has been incorporated into polymer nanoparticles with a mitochondrial targeting moiety. Upon ROS generation, camptothecin is released from modified PEG with thioketal linkers. Mitochondria targeting is achieved by modifying the surface of the particles with triphenylphosphine cations. Testing in NCI-H460 (lung derived) tumours in mice showed an 80% reduction in tumour size after irradiation with 633 nm light [125]. Nano metal organic frameworks have also been used as a means of delivering and increasing the photosensitizer effect of 5,15-di(*p*methylbenzoato)chlorin (DBC-UiO). Hf_6_(u_3_-O)_4_(u_3_-OH)_4_ frame points and DBC-UiO linkers were used to make up the framework of the material, and the linkers also act as a photosensitising agent, able to convert triplet oxygen to singlet oxygen under visible light wavelengths and cause cell death by oxidative stress. These nano metal organic frameworks showed high efficiencies in treating xenografted colon cancer in mice with PDT [126]. Manganese ferrite nanoparticles anchored to the surface of mesoporous silica nanoparticles have been used as another method to overcome the problems posed by hypoxia in conventional PDT. Using the Fenton reaction (H_2_O_2_ conversion into O_2_ by an Fe catalyst) [127] and the high concentration of H_2_O_2_ in cancer microenvironments, these nanoparticles can produce a continuous source of O_2_ within tumours. The oxygen production allows the Ce6 photosensitizer loaded in the silica nanoparticles to increase ROS generation and thus avoid hypoxia [128]. In addition, iron oxide nanocubes have been used to create an innovative combined photothermal and magneto- thermal treatment. In aqueous suspension under a NIR light of 808 nm and an AMF, specific loss heating powers of 5000 W/g were observed, 15-fold amplification of the inductive heating effect alone. In SKOV3, PC3 and A431 cells complete tumour regression was seen in vivo [129]. Although this method of dual heating shows remarkable ability to induce massive apoptosis in tumour tissue, the capacity to damage healthy tissue remains a huge drawback. 

**Figure 6:**
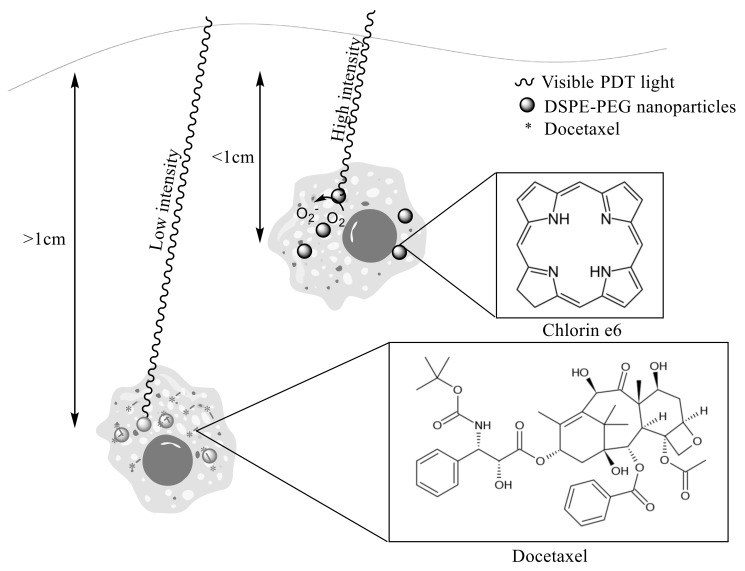
Cell death mechanisms utilising different intensities of near infrared radiation (NIR) light for treating tumours at differing depths. At low penetration depths cell death is caused by reactive oxygen species (ROS) generated by Ce6 as photodynamic therapy (PDT). At high penetration depths and low light intensity cell death is caused by the release of docetaxel.

To add to the functionality of the nanoparticulate systems, nucleotide sequences can be incorporated. One example of a nucleotide-incorporating smart release system has been developed to treat triple-negative breast cancer. Polymeric (DSPE-PEG) nanoparticles containing docetaxel as a chemotherapeutic agent, anti-twist siRNA as a gene therapy agent, and chlorin e6 (Ce6) as a photosensitizer have been created (Figure 6). At low penetration depths photodynamic therapy as a result of the Ce6 photosensitizer is responsible for most of the cell death. However, under conditions mimicking deep tissue tumours and low light conditions, chemotherapy and gene therapy facilitated cell death. Here the siRNA was incorporated to silence the TWIST gene which promotes epithelial-mesenchymal transition and metastasis. Light-assisted endolysosomal escape assisted the activation of chemo and gene therapy [115]. 

A smart light responsive drug delivery complex enabling the selective release of doxorubicin and HER2-targeted lapatinib from gold shelled silica NPs has been created. Doxorubicin was bound to the surface of the core-shell nanoparticles by conjugation to thiolated dsDNA. Heating of the gold using NIR causes a breakdown of the thiol bonds and releases the drug. Additionally, lapatinib was conjugated to immobilized human serum albumin which could be released by the photothermal effect [130]. This demonstrated a synergistic effect between hyperthermia and activated drug delivery, cytotoxicity in breast cancer cell lines, and also demonstrated a means of loading many hydrophobic drug molecules. DNA has also been used to mask aptamers to give another layer of targeting specificity. Single walled carbon nanotubes containing doxorubicin were capped with sgc8 targeting aptamers and a complementary DNA sequence to hybridize to the aptamers. Photothermal heating with NIR of the nanotubes by NIR dehybridizes double stranded DNA unmasking the aptamers allowing cancer specific cell targeting and binding [131].

#### 2.3.3. X-ray Activation 

In addition to visible and NIR light, X-ray induced PDT was demonstrated for the first time in 2015. Nanoparticles consisting of an Sr_2_Al_2_O_4_:Eu^2+^ upconverting core and a mesoporous silica membrane containing the photosensitizer MC540 have been shown to cause efficient tumour shrinkage under X-ray stimulation. U-87 MG (glioblastoma) tumour bearing mice were treated at tumour depths of several centimetres, which would not be possible by conventional PDT. The mice also exhibited negligible toxicity in vital organs, highlighting the wide therapeutic index of this treatment [132]. Unlike NIR and visible light, X-rays can penetrate deep into tissue, removing the limit of treatment from surface dwelling tumours. In addition to this, the X-rays used for stimulation can be achieved with existing hospital equipment and tuned to give almost no off-target damage. Fluorodeoxyglucose (FDG), the most commonly used radiotracer in PET has also been used as a source of photoelectronic energy. Nano micelles were prepared containing the photosensitizer titanocene, to target the GLUT1 glucose transporter protein. Delivery of FDG alongside nano micelles into metastatic breast cancer cell lines (PyMT-TC) resulted in considerable cell death. However, circulation half-life of nano micelles is very short <2 h and so its utility in the clinic may be limited. Unfortunately, resistance in small clusters of cancer cells was also seen [133]. 

## 3. Evaluation of Both Intrinsic and Extrinsic Activation Systems

Activatable systems have the potential to provide an even greater level of specificity to cancer treatments. The aim will be to provide treatment with fewer side-effects and to enable a greater dose at the target site. While exploitation of the intrinsic changes in the physiology of cancer cells is an attractive proposal, problems lie in the relative changes seen compared to healthy cells. Enzyme targets, for example, are often expressed to some level in normal cells and can therefore lead to non-specific release. However, despite this, there are concentration differences in enzymes, proteins, and other small molecules which may assist in altering the therapeutic index by selective activation. In a similar fashion, pH-targeting may be able to provide an improved degree of resolution, but is reliant on either being targeted to the tumour, or intracellular uptake specifically in cancer cells. This can be improved by the addition of targeting moieties or combination with other functionalities such as directed radiotherapy or ultrasound. Despite this, those systems carrying chemotherapeutics or nucleic acids which are only released at the site of action can prevent degradation of the cargo, ensuring that more of the active compound is available for therapy. 

Applications which use extrinsic activation are increasingly used to target therapies. Ultrasound has been used for many years in medicine for imaging, and may now find a new role. Ultrasound has been shown to have negligible cytotoxicity. However, there are limitations due to the poor penetration of ultrasound through bone and air. This means that activation in regions such as the lungs or ribs could be difficult. More critically, some ultrasound frequencies cannot be used to treat intracranial tumours due to the possibility of inertial cavitation causing petechial haemorrhage [74].

Sonoporation also has limitations due to the fact that increased poration allows accumulation of therapeutic molecules in undesired tissues and organs, especially when performed close to vascular tissue [71]. This would be especially damaging when administering highly cytotoxic drugs. Ultimately this contradicts the selective uptake advantage of using ultrasound but could be overcome by optimization of ultrasound equipment. However, one big advantage for ultrasound therapy is that the frequencies and amplitudes needed for activation are achievable through existing hospital equipment. 

Activation using NIR light has provided a means of stimulus that is a cheap, safe and minimally adverse to healthy tissue. However, penetration depths are still sub 4 mm through skin [134], which limits the usefulness of this technique. This is even more of an issue for wavelengths of light in the visible range. New systems using X-ray energy to activate PDT may be able to address the issue of limited utility due to penetration depth. An alternative technique which is highly penetrating, but non-ionizing, involves the use of AMF. Its ability to access areas of the body where other stimuli often fail is a distinct advantage. Also, AMF can be focused in targeted areas of the body with minimal adverse effects to the surrounding tissue. 

While many activatable therapies are in their infancy, and are yet to reach the clinic, it can be clearly seen that there are many advantages to such systems. The ability to release therapies specifically at the target site can only confer advantages for cancer patients.
